# Chest CT Severity Score and Systemic Inflammatory Biomarkers as Predictors of the Need for Invasive Mechanical Ventilation and of COVID-19 Patients’ Mortality

**DOI:** 10.3390/diagnostics12092089

**Published:** 2022-08-29

**Authors:** Ioana Halmaciu, Emil Marian Arbănași, Réka Kaller, Adrian Vasile Mureșan, Eliza Mihaela Arbănași, Nicolae Bacalbasa, Bogdan Andrei Suciu, Ioana Iulia Cojocaru, Andreea Ioana Runcan, Florin Grosu, Vlad Vunvulea, Eliza Russu

**Affiliations:** 1 Department of Radiology, Mureș County Emergency Hospital, 540136 Targu-Mures, Romania; 2 Department of Anatomy, George Emil Palade University of Medicine, Pharmacy, Science, and Technology of Targu Mures, 540139 Targu-Mures, Romania; 3 Clinic of Vascular Surgery, Mureș County Emergency Hospital, 540136 Targu-Mures, Romania; 4 Department of Surgery, George Emil Palade University of Medicine, Pharmacy, Science, and Technology of Targu Mures, 540139 Targu-Mures, Romania; 5 Faculty of Pharmacy, George Emil Palade University of Medicine, Pharmacy, Science, and Technology of Targu Mures, 540139 Targu-Mures, Romania; 6 Department of Obstetrics and Gynecology, “Carol Davila” University of Medicine and Pharmacy, 020021 Bucharest, Romania; 7 First Clinic of Surgery, Mureș County Emergency Hospital, 540136 Targu-Mures, Romania; 8 Department of Histology, Lucian Blaga University of Sibiu, 550169 Sibiu, Romania

**Keywords:** total system score, COVID-19, MLR, NLR, SII, SIRI, AISI, IL-6

## Abstract

Background: Numerous tools, including inflammatory biomarkers and lung injury severity scores, have been evaluated as predictors of disease progression and the requirement for intensive therapy in COVID-19 patients. This study aims to verify the predictive role of inflammatory biomarkers [monocyte to lymphocyte ratio (MLR), neutrophil to lymphocyte ratio (NLR), systemic inflammatory index (SII), Systemic Inflammation Response Index (SIRI), Aggregate Index of Systemic Inflammation (AISI), and interleukin-6 (IL-6)] and the total system score (TSS) in the need for invasive mechanical ventilation (IMV) and mortality in COVID-19 patients. Methods: The present study was designed as an observational, analytical, retrospective cohort study and included all patients over 18 years of age with a diagnosis of COVID-19 pneumonia, confirmed through real time-polymerase chain reaction (RT-PCR) and radiological chest CT findings admitted to County Emergency Clinical Hospital of Targu-Mureș, Romania, and Modular Intensive Care Unit of UMFST “George Emil Palade” of Targu Mures, Romania between January 2021 and December 2021. Results: Non-Survivors patients were associated with higher age (*p* = 0.01), higher incidence of cardiac disease [atrial fibrillation (AF) *p* = 0.0008; chronic heart failure (CHF) *p* = 0.01], chronic kidney disease (CKD; *p* = 0.02), unvaccinated status (*p* = 0.001), and higher pulmonary parenchyma involvement (*p* < 0.0001). Multivariate analysis showed a high baseline value for MLR, NLR, SII, SIRI, AISI, IL-6, and TSS independent predictor of adverse outcomes for all recruited patients. Moreover, the presence of AF, CHF, CKD, and dyslipidemia were independent predictors of mortality. Furthermore, AF and dyslipidemia were independent predictors of IMV need. Conclusions: According to our findings, higher MLR, NLR, SII, SIRI, AISI, IL-6, and TSS values at admission strongly predict IMV requirement and mortality. Moreover, patients above 70 with AF, dyslipidemia, and unvaccinated status highly predicted IMV need and fatality. Likewise, CHF and CKD were independent predictors of increased mortality.

## 1. Introduction

Due to the rapid spread of the SARS-Cov-2 (severe respiratory syndrome coronavirus 2) infection, the World Health Organization (WHO) declared the outbreak of the COVID-19 pandemic on 11 March 2020, which has become a global phenomenon and public health problem, which in the last two years had a negative impact on current medical practice [1,2,3]. Despite the development of antiviral therapies, severe forms of the disease require intensive therapy and have a high mortality rate [4,5]. Real-Time PCR (RT-PCR) is the preferred technique since it provides the most accurate disease diagnosis [6,7].

COVID-19 patients’ symptoms might vary from mild to severe, ranging from fever, headache, and loss of taste and smell, to acute respiratory distress syndrome (ARDS), thromboembolic events, cardiac injury, or sepsis [8,9,10,11,12,13,14,15].

Numerous tools, including inflammatory biomarkers and lung injury severity scores, have been evaluated as predictors of disease progression and the requirement for intensive therapy in COVID-19 patients [16,17,18,19,20,21,22,23,24,25,26].

Chest computer tomography (CT) is a non-invasive, quick imaging tool that plays an essential role in the diagnosis and progression of COVID-19 patients [27,28,29,30]. Numerous scores have been established for evaluating the degree of pulmonary damage and standardized radiological interpretation [31,32,33,34]. Among these is the Total Score System (TSS), introduced by Chung et al. [35], whose prognostic role was studied and established for the negative progression, as well as the degree of severity and mortality, of COVID-19 patients [31,36,37].

Inflammation is a major factor in the evolution of severe COVID-19 variants. It is well known that a strong inflammatory response compromises the immune system; therefore, assessing systemic biomarkers of inflammation can provide extra information to diagnose and stratify the severity of the disease. Apart from well-known biomarkers of inflammation such as C-reactive protein, interleukin-6 (IL-6), and procalcitonin, hematological indices have gained popularity in recent years in the specialized literature due to their inexpensive cost and performance [7,19,20,21,22,23,24,25,26,38,39,40,41]. Moreover, hematological indices such as neutrophil-lymphocyte ratio (NLR), monocyte-lymphocyte ratio (MLR), systemic immune inflammation index (SII), Systemic Inflammation Response Index (SIRI), and Aggregate Index of Systemic Inflammation (AISI), have been applied to predict the prognosis in patients with the cardiovascular disease [42,43,44], acute limb ischemia [45,46], chronic kidney disease [47,48], peripheral artery disease [49], malignancy [50,51,52], and more recently in the case of COVID-19 patients [7,19,20,21,22,23,24,25,26].

This study aims to verify the predictive role of inflammatory biomarkers (MLR, NLR, SII, SIRI, AISI, and IL-6) and the TSS in need of invasive mechanical ventilation (IMV) and mortality in COVID-19 patients.

## 2. Materials and Methods

### 2.1. Study Design

The present study was designed as an observational, analytical, retrospective cohort study and included all patients over 18 years of age with a diagnosis of COVID-19 pneumonia, confirmed by real-time-polymerase chain reaction (RT-PCR) and radiological chest CT findings, admitted to County Emergency Clinical Hospital of Targu-Mureș, Romania, and Modular Intensive Care Unit of UMFST “George Emil Palade” of Targu Mures, Romania between January 2021 and December 2021. Exclusion criteria were as follows: patients who died and need invasive mechanical ventilation in the first 24 h from admission, patients with end-stage kidney disease and dialysis, recent malignancy diagnosed within a maximum of six months prior to our studied period, and any leukemia or other hematological disorders, major surgery: any major resection or reconstruction of any digestive organ, cardiovascular reconstruction/revascularizations (major heart/aortic surgeries), major surgery of the lungs or kidneys, autoimmune diseases, and patients without a chest CT scan in the first 24 h.

Patients included in the study were initially divided into two groups depending on their poor outcome during the hospitalization named “Survivors” and “non-Survivors.” The ideal cut-off value for MLR, NLR, SII, SIRI, AISI, IL-6 and TSS was used to calculate the need for IMV and mortality.

### 2.2. Data Collection

The patients’ demographic data were extracted from the hospital’s electronic database. We searched for the following comorbidities in the medical history: arterial hypertension (AH), ischemic heart disease (IHD), atrial fibrillation (AF), chronic heart failure (CHF), myocardial infarction (MI), type 2 diabetes (T2D), chronic obstructive pulmonary disease (COPD), peripheral arterial disease (PAD), chronic kidney disease (CKD), cerebrovascular accident (CVA), dyslipidemia, tobacco use, obesity, and length of hospital stay. in addition, we collected data from the first blood test result (hemoglobin, hematocrit, neutrophil count, lymphocyte count, monocyte count, platelet count, IL-6, glucose level, cholesterol level, triglyceride level, potassium level, blood urea nitrogen level, and creatinine level).

### 2.3. Systemic Inflammatory Markers

The systemic inflammation index was determined from the first blood test result. The MLR, NLR, SII, SIRI, and AISI were calculated using the equations below:

$$MLR=\frac{total\;number\;of\;monocytes}{total\;number\;of\;lymphocytes}$$

$$NLR=\frac{total\;number\;of\;neutrophils}{total\;number\;of\;lymphocytes}$$

$$SII=\frac{total\;number\;of\;neutrophils\;\times \;total\;number\;of\;platelets}{total\;number\;of\;lymphocytes}$$

$$SIRI=\frac{total\;number\;of\;neutrophils\;\times \;total\;number\;of\;monocytes}{total\;number\;of\;lymphocytes}$$

$$AISI=\frac{total\;number\;of\;neutrophils\;\times \;total\;number\;of\;platelets\;\times \;total\;number\;of\;monocytes}{total\;number\;of\;lymphocytes}.\;$$

### 2.4. Chest CT Severity Score

Chest CT exams were performed in the first 24 h from admission. Image analysis was performed using a PACS (Picture Archiving and Communication System) workstation (INFINITT Healthcare Co., Ltd., Seoul, Korea). Chest CT images were assessed to evaluate the extent of pulmonary parenchymal involvement for the presence of ground-glass opacities (GGOs), consolidation, and pleural effusion.

TSS was calculated by quantifying the disease-affected areas for each lobe to evaluate pulmonary parenchymal involvement. Each of the five lobes was given a score ranging from 0 to 4, based on the percentage of the affected area as none (0%), minimal (1–25%), mild (26–50%), moderate (51–75%), or severe (76–100%). TSS was calculated by adding the values for five lobes ranging from 0 to 20.

### 2.5. Vaccination Status

During the studied period in Romania, four different vaccines were used Pfizer (BioNTech, Mainz, Germany), AstraZeneca (Oxford University, Oxford, UK), Moderna (National Institute of Allergy and Infectious Diseases and Biomedical Advanced Research and Development Authority, Cambridge, MA, USA), and Janssen (Johnson and Johnson, New Brunswick, NJ, USA). Depending on the number of doses for each type of vaccine, patients were registered as unvaccinated, partially vaccinated, and fully vaccinated.

### 2.6. Study Outcomes

The primary endpoints were the need for IMV, in-hospital mortality rate, and a composite endpoint of IMV need and mortality. Outcomes were stratified for the baseline’s optimal MLR, NLR, SII, SIRI, AISI, IL-6, and TSS cut-off value.

### 2.7. Statistical Analysis

SPSS for Mac OS version 28.0.1.0 was used for statistical analysis (SPSS, Inc., Chicago, IL). Chi-square tests were used to assess the associations of MLR, NLR, SII, SIRI, AISI, IL-6, and TSS with category factors, while t-Student or Mann–Whitney tests were used to assess differences in continuous variables. To assess the predictive power and establish cut-off MLR, NLR, SII, SIRI, AISI, IL-6, and TSS, the receiver operating characteristic (ROC) curve analysis was utilized. The receiver operating characteristic (ROC) curve analysis was used to determine the appropriate MLR, NLR, SII, SIRI, AISI, IL-6, and TSS cut-off values based on the Youden index (Youden Index = Sensitivity + Specificity − 1, ranging from 0 to 1). To identify independent predictors of IMV need, mortality, and a composite endpoint of IMV need and mortality, a multivariate logistic regression analysis using variables with *p* < 0.1 was undertaken.

## 3. Results

During the study period, 267 patients diagnosed with COVID-19 met the inclusion criteria and followed up during hospitalization. The mean age was 71.19 ± 10.25 (33–94), and 159 patients were male (59.55%) (Table 1). During the hospitalization, 60 patients (22.47%) needed IMV, 82 patients died (30.71%), and 45 patients (16.85%) needed IMV and deceased later, respectively. Depending on the survival status during the hospitalization, the patients were enrolled in two groups: Survivors and Non-Survivors. Mean age was statistically higher in the second group (*p* = 0.01). In terms of comorbidities and risk factors, in the non-Survivors group was a higher incidence of AF (*p* = 0.0008), CHF (*p* = 0.01), dyslipidemia (*p* = 0.01), and CKD (*p* = 0.002). Regarding the Pulmonary CT scan findings, in the second group, all five pulmonary lobes were affected in a higher proportion (*p* < 0.0001), and the TSS was higher (*p* < 0.0001). Regarding vaccination status, the non-Survivors group had a higher incidence of unvaccinated (*p* = 0.001) and a lower incidence of fully vaccinated (*p* = 0.0005). Moreover, several variables from Laboratory data were associated with poor outcomes: non-Survivors had lower lymphocyte (*p* < 0.0001) and potassium level (*p* < 0.0001), and higher neutrophils (*p* < 0.0001), monocyte (*p* = 0.0006), glucose (*p* < 0.0001), MLR (*p* < 0.0001), NLR (*p* < 0.0001), SII (*p* < 0.0001), SIRI (*p* < 0.0001), AISI (*p* < 0.0001), and IL-6 (*p* < 0.0001). In addition, the non-Survivors patients had a higher incidence of IMV need (*p* < 0.0001) and a long hospital stay (*p* = 0.0005). The rest of the comorbidities and laboratory data are presented in Table 1.

**Figure 1:**
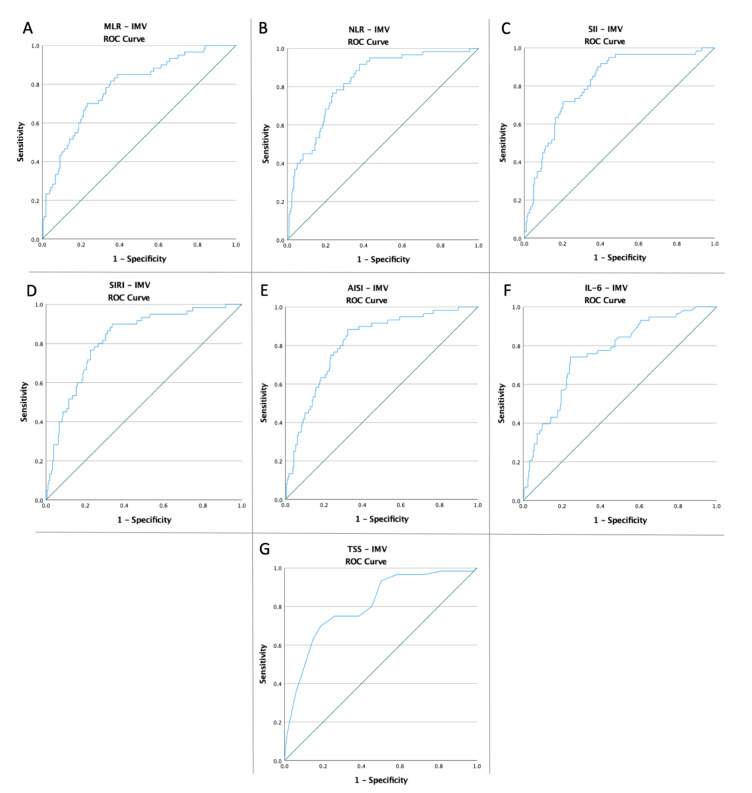
ROC curve analysis concerning the IMV need (**A**) MLR (AUC: 0.829; *p* < 0.0001), (**B**) NLR (AUC: 0.856; *p* < 0.0001), (**C**) SII (AUC: 0.858; *p* < 0.0001), (**D**) SIRI (AUC: 0.785; *p* < 0.0001), (**E**) AISI (AUC: 0.765; *p* < 0.0001), (**F**) IL-6 (AUC: 0.762; *p* < 0.0001), and (**G**) TSS (AUC: 0.759; *p* < 0.0001).

**Figure 2:**
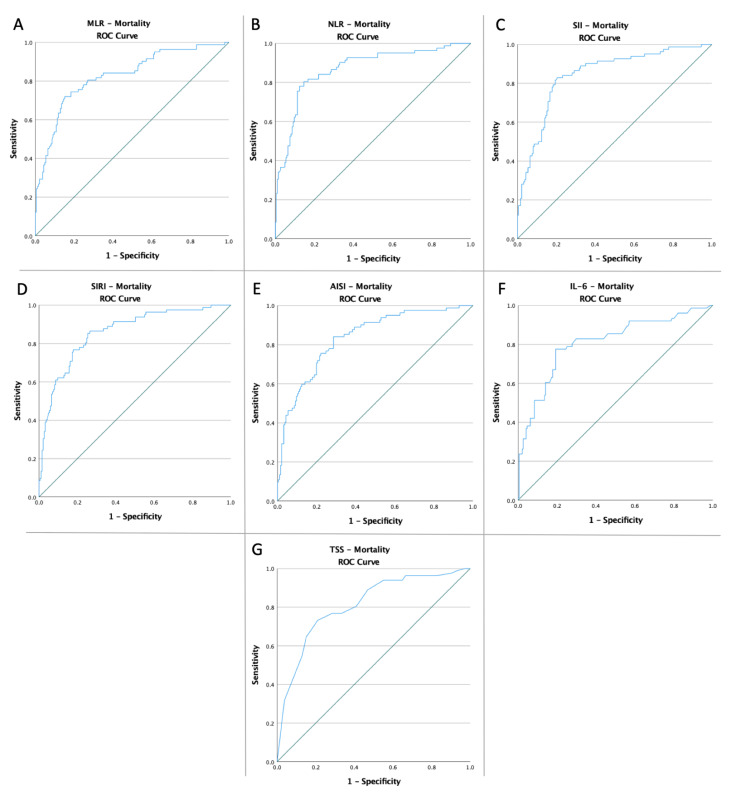
ROC curve analysis concerning the mortality (**A**) MLR (AUC: 0.829; *p* < 0.0001), (**B**) NLR (AUC: 0.856; *p* < 0.0001), (**C**) SII (AUC: 0.858; *p* < 0.0001), (**D**) SIRI (AUC: 0.785; *p* < 0.0001), (**E**) AISI (AUC: 0.765; *p* < 0.0001), (**F**) IL-6 (AUC: 0.808; *p* < 0.0001), and (**G**) TSS (AUC: 0.759; *p* < 0.0001).

**Figure 3:**
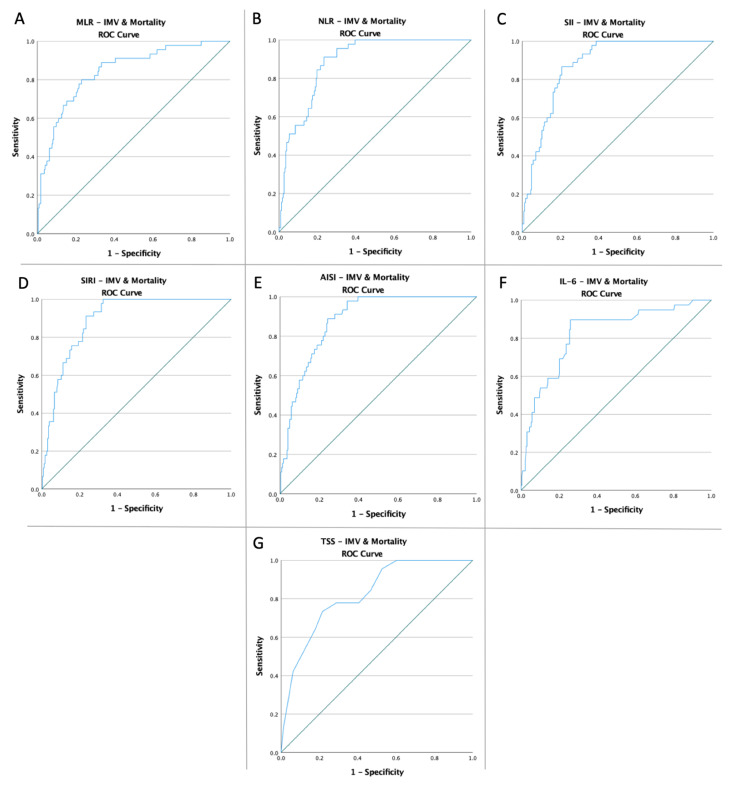
ROC curve analysis concerning the IMV need and mortality (**A**) MLR (AUC: 0.829; *p* < 0.0001), (**B**) NLR (AUC: 0.856; *p* < 0.0001), (**C**) SII (AUC: 0.858; *p* < 0.0001), (**D**) SIRI (AUC: 0.785; *p* < 0.0001), (**E**) AISI (AUC: 0.765; *p* < 0.0001), (**F**) IL-6 (AUC: 0.825; *p* < 0.0001), and (**G**) TSS (AUC: 0.759; *p* < 0.0001).

Receiver operating characteristic curves of MLR, NLR, SII, SIRI, AISI, IL-6, and TSS were created to determine whether the baseline of these markers was predictive of IMV need, mortality, and common endpoint in patients with COVID-19 (Figure 1, Figure 2 and Figure 3). The optimal cut-off value obtained from Youden’s index, areas under the curve (AUC), and the predictive accuracy of the markers are listed in Table 2.

Depending on the optimal cut-off value according to the ROC, the outcomes were further analyzed after dividing the patients into paired groups. There was a higher incidence of all adverse outcomes for all the markers analyzed, as seen in Table 3.

Multivariate analysis showed that a high baseline value for all the analyzed markers was an independent predictor of adverse outcomes for all recruited patients. Furthermore, for all hospitalized patients, an age over 70 (*p* = 0.02; *p* = 0.001; *p* = 0.005), AF (*p* = 0.009; *p* < 0.0001; *p* = 0.01), dyslipidemia (*p* = 0.01; *p* = 0.01; *p* = 0.02), and unvaccinated (*p* = 0.04; *p* < 0.001; *p* = 0.002) was an independent predictor of a poor prognosis for all the outcomes. CHF and CKD were independent predictors for mortality (*p* = 0.01) and composite endpoint (*p* = 0.02) but not for IMV need (Table 4).

## 4. Discussion

This study included 267 individuals diagnosed with COVID-19 pneumonia. We determined the preoperative values for all patients for inflammatory biomarkers and TSS and monitored IMV requirement, mortality rate, and a composite endpoint of IMV need and mortality. The most important finding of our study is that a high baseline value for MLR, NLR, SII, SIRI, AISI, and TSS (*p* < 0.0001) is a strong predictor of all outcomes. Moreover, to the best of our knowledge, our study demonstrates for the first time that patients with higher MLR, NLR, SII, SIRI, AISI, and TSS showed a higher risk of disease progression to IMV need and intra-hospital mortality.

According to our study, the elderly patients are associated with IMV need (OR: 1.97; 95% CI: 1.07–3.63; *p* = 0.02), and higher mortality (OR: 2.49; 95% CI: 1.43–4.35; *p* = 0.001). Moreover, as numerous published studies have demonstrated, age is associated with a negative disease evolution and, more frequently, ICU admission [53,54,55]. Similar to our study, the presence of cardiac pathologies, chronic kidney disease, and risk factors such as dyslipidemia increases the probability of developing severe forms of COVID-19 [56,57,58,59,60,61], requirements for IMV [56,57,58,59,60,61], and mortality [57,58,59,60,61].

Similar to our research, Bellos et al. [36] discovered that a TSS higher than 10.5 (75% Sensitivity, 70% Specificity; AUC:0.811) is a prognostic factor for ICU admission (12.60 ± 4.25 vs. 7.38 ± 4.23, *p*: 0.004). Furthermore, Li et al. [31] published research with 78 patients in which they established that high TSS levels over 7.5 (AUC:0.918; 82.6% Sensitivity 100% Specificity; *p* < 0.001) are associated with the severe form of the disease. S.M.H. Tabatabaei et al. [62], Zhou et al. [63], and Tharwat el al. [64] found that high TSS values are predictive of COVID-19 patient mortality [(OR:1.99; 95% CI: 1.01–4.06; *p* = 0.04), (OR:6.87; 95% CI: 2.13–22.17; *p* = 0.001), and (OR:2.08; 95% CI: 1.57–2.74; *p* < 0.001)].

Regarding hematological indices, NLR and SII have been analyzed in numerous articles published recently [19,22,23,56,65,66]. Thus, Moisa et al. [56] demonstrated that a basal value of NLR > 11 (HR:4.6; 95% CI:2.80–7.56; *p* < 0.001), MLR >0.64 (HR:2.38; 95% CI:1.70–3.33; *p* < 0.001), and SII > 3700 (HR:2.44; 95% CI:1.68–3.54; *p* < 0.001) are independent prognostic factors of mortality for the 272 patients diagnosed with COVID-19 [56]. Moreover, Citu et al. discovered the association of high values of NLR > 9.1 (HR:3.85; 95% CI:1.35–10.95; *p* = 0.01), and MLR > 0.69 (HR:3.05; 95% CI:1.16–8.05; *p* = 0.02), and an increased mortality rate [22].

Similarly, Kudlinsky et al. published an article in which they proved the prognostic impact of NLR > 11.57 (*p* = 0.0008) and SII > 2058 (*p* = 0.02) in COVID-19 patients’ death [65]. In a cohort study involving 411 COVID-19 patients, Regolo et al. discovered a correlation between baseline NLR values greater than 11.38 and the necessity for ICU admission (*p* < 0.0001) [19]. Moreover, in the papers conducted by Hamad et al. [65], Nalbant et al. [66], and Fois et al. [55], high values of SIRI and AISI were related to the severe form of the disease, necessity of ICU, and increased mortality [55].

The results of our study are in agreement with those recently published in the literature, thus the high values of MLR (>0.54) (OR:6.44; 95% CI: 3.42–12.13; *p* < 0.001; and OR:6.49; 95% CI: 2.51–22.24; *p* < 0.001)], NLR (>6.82 and >6.97) (OR:10.59; 95% CI: 5.37–20.88; *p* < 0.001; and OR:24.13; 95% CI: 12.20–47.73; *p* < 0.001), SII (>2166.04 and >1739.36) (OR:9.93; 95% CI: 5.15–19.14; *p* < 0.001; and OR:18.78; 95% CI: 9.54–36.97; *p* < 0.001), SIRI (>3.66 and >3.84) (OR:17.16; 95% CI: 7.22–42.49; *p* < 0.001; and OR:17.91; 95% CI: 8.77–36.58; *p* < 0.001), AISI (>994.76 and >973.59) (OR:15.82; 95% CI: 6.86–36.65; *p* < 0.001; and OR:13.21; 95% CI: 6.74–25.90; *p* < 0.001), IL-6 (>30.95 and >28.17) (OR:8.88; 95% CI: 4.55–17.30; *p* < 0.001; and OR:14.45; 95% CI: 7.55–27.61; *p* < 0.001), as well as high TSS values (>15.5 and >16.5) (OR:8.50; 95% CI: 4.38–16.47; *p* < 0.001; and OR:10.24; 95% CI: 5.59–18.77; *p* < 0.001) are independent factors for predicting the need for IMV and in-hospital mortality, in the case of COVID-19 patients.

Given the findings of our study, which support the work published in the literature during the last two years, as well as the low cost and ease of use of hematological markers and the lung damage score, their use in medical practice allows for better stratification of risk groups and the establishment of appropriate therapeutic management, thus improving the progression of patients with COVID-19.

Our study has certain limitations, despite the statistically significant results. First and foremost, it is a retrospective, monocentric research with patient follow-up during hospitalization. Prospective multicenter trials with long-term follow-ups are recommended in the future. Furthermore, due to the study’s retrospective nature, we could not access data about chronic medications used before admission (such as corticosteroids and anti-inflammatories meds). Therefore, we could not establish the effect of other medications on inflammatory biomarkers. Furthermore, additional research is necessary to support our findings.

## 5. Conclusions

According to our findings, higher MLR, NLR, SII, SIRI, AISI, IL-6, and TSS values at admission strongly predict IMV requirement and mortality. Moreover, patients above 70 with AF, dyslipidemia, and unvaccinated status highly predicted IMV need and fatality. Likewise, CHF and CKD were independent predictors of increased mortality. Given the ease of access and low cost of these ratios and chest CT severity score, they can be used for admission risk group categorization, improved patient care, and the development of predictive patterns.

## Figures and Tables

**Table 1:** Demographic data, comorbidities, risk factors, chest CT findings, laboratory findings, and outcomes for all patients and the two subgroups were divided according to the poor outcomes.

Variables	All Patients *n* = 267	Survivors *n* = 185	Non-Survivors *n* = 82	*p* Value (OR; CI 95%)
Age mean ± SD (min-max)	71.19 ± 10.25 (33–94)	70.01 ± 8.99 (46–91)	73.85 ± 12.29 (33–94)	0.01
Male sex no. (%)	159 (59.55%)	112 (60.54%)	47 (57.32%)	0.62 (0.87; 0.51–1.48)
**Comorbidities & Risk Factors**				
AH, no. (%)	167 (62.55%)	116 (62.70%)	51 (62.20%)	0.93 (0.97; 0.57–1.67)
IHD, no. (%)	145 (54.31%)	97 (52.43%)	48 (58.54%)	0.35 (1.28; 0.75–2.16)
AF, no. (%)	79 (29.59%)	43 (23.24%)	36 (43.90%)	0.0008 (2.58; 1.48–4.49)
CHF, no. (%)	130 (48.69%)	81 (43.78%)	49 (59.76%)	0.01 (1.90; 1.12–3.23)
MI, no. (%)	80 (29.96%)	50 (27.03%)	30 (36.59%)	0.11 (1.55; 0.89–2.71)
T2D, no. (%)	116 (43.45%)	82 (44.32%)	34 (41.46%)	0.66 (0.88; 0.52–1.50)
COPD, no. (%)	62 (23.22%)	44 (23.78%)	18 (21.95%)	0.74 (0.90; 0.48–1.68)
Dyslipidemia, no. (%)	150 (56.18%)	95 (51.35%)	55 (67.07%)	0.01 (1.92; 1.12–3.32)
PAD, no. (%)	120 (44.94%)	85 (45.95%)	35 (42.68%)	0.62 (0.87; 0.51–1.48)
CKD, no. (%)	57 (21.35%)	30 (16.22%)	27 (32.93%)	0.002 (2.54; 1.38–4.64)
CVA, no. (%)	76 (28.46%)	46 (24.86%)	30 (36.59%)	0.051 (1.74; 0.99–3.05)
Obesity, no. (%)	69 (44.94%)	49 (26.49%)	20 (24.39%)	0.71 (0.89; 0.49–1.63)
Tobacco, no. (%)	99 (37.08%)	68 (36.76%)	31 (37.80%)	0.87 (1.04; 0.61–1.78)
**Chest CT Findings**				
Consolidation, no. (%)	95 (35.58%)	62 (33.51%)	33 (40.24%)	0.29
Pleural Effusion, no. (%)	38 (14.23%)	26 (14.05%)	12 (14.63%)	0.90
Ground Glass-Opacities, no. (%)	167 (62.55%)	114 (61.62%)	53 (64.63%)	0.63
Right Upper Lobe, mean ± SD	2.30 ± 1.19	1.97 ± 1.15	3.04 ± 0.94	<0.0001
Right Middle Lobe, mean ± SD	2.58 ± 1.29	2.25 ± 1.29	3.32 ± 0.96	<0.0001
Right Lower Lobe, mean ± SD	2.84 ± 1.15	2.54 ± 1.14	3.52 ± 0.83	<0.0001
Left Upper Lobe, mean ± SD	2.12 ± 1.10	1.79 ± 1.02	2.85 ± 0.93	<0.0001
Left Lower Lobe, mean ± SD	2.74 ± 1.17	2.40 ± 1.16	3.51 ± 0.75	<0.0001
Total System Score. mean ± SD	12.57 ± 5.26	10.95 ± 5.07	16.24 ± 3.78	<0.0001
**Vaccination Status**				
**UNVACCINATED**, no. (%)	69 (25.84%)	37 (20%)	32 (39.02%)	0.001
**PARTIALLY VACCINATED**, no. (%)	54 (20.22%)	35 (18.91%)	19 (23.17%)	0.42
**FULLY VACCINATED**, no. (%)	144 (53.93%)	113 (61.08%)	31 (37.80%)	0.0005
**Laboratory Data**				
Hemoglobin g/dL, median [Q1–Q3]	12.51 [10.73–13.9]	12.56 [10.7–13.81]	12.50 [10.96–14.2]	0.21
Hematocrit %, median [Q1–Q3]	38.99 [32.74–42.75]	38.4 [32.5–42.3]	39.1 [33.32–44.5]	0.10
Neutrophils ×10^3^/uL, median [Q1–Q3]	7.6 [5.86–10.93]	6.82 [5.27–8.95]	10.59 [7.50–13.73]	<0.0001
Lymphocytes ×10^3^/uL, median [Q1–Q3]	1.58 [1.09–2.09]	1.79 [1.41–2.26]	1.05 [0.63–1.41]	<0.0001
Monocyte ×10^3^/uL, median [Q1–Q3]	0.64 [0.46–0.88]	0.61 [0.46–0.81]	0.73 [0.56–1.08]	0.0006
PLT ×10^3^/uL, median [Q1–Q3]	257 [207.05–318]	257 [212–314.5]	257.5 [206–338.85]	0.43
Glucose mg/dL, median [Q1–Q3]	118 [97–149.5]	107 [95–139.5]	139 [116.02–175.12]	<0.0001
Cholesterol mg/dL, median [Q1–Q3]	177.7 [144.25–212.7]	179.2 [144.9–214.4]	164.95 [143.6–205.47]	0.13
Triglyceride mg/dL, median [Q1–Q3]	114.8 [91.3–166.95]	114.8 [92.7–160]	113.95 [88.32–169.7]	0.49
Potassium mmol/L, median [Q1–Q3]	4.59 [4.09–5.37]	4.79 [4.3–5.49]	4.18 [3.77–4.99]	<0.0001
Sodium mmol/L, median [Q1–Q3]	140 [139–141]	140 [139–141]	140 [139–142]	0.11
BUN mg/dL, median [Q1–Q3]	43.6 [33–56.05]	42.4 [33.3–54.7]	46.55 [32.55–67.8]	0.10
Creatinine mg/dL, median [Q1–Q3]	0.94 [0.75–1.15]	0.94 [0.75–1.14]	0.92 [0.78–1.23]	0.25
MLR, median [Q1–Q3]	0.40 [0.27–0.67]	0.33 [0.24–0.47]	0.75 [0.51–1.25]	<0.0001
NLR, median [Q1–Q3]	4.90 [2.88–9.79]	3.73 [2.61–5.78]	11.04 [7.77–18.24]	<0.0001
SII, median [Q1–Q3]	1408.12 [721.44–2464.28]	1012.58 [618.39–1599.85]	2613.55 [1950.20–5024.20]	<0.0001
SIRI, median [Q1–Q3]	3.03 [1.68–7.27]	2.21 [1.41–4.04]	9.13 [5.10–12.76]	<0.0001
AISI, median [Q1–Q3]	856.54 [416.97–2224.48]	594.41 [350.51–1180.64]	2349.60 [1310.65–3817.96]	<0.0001
**IL-6**, median [Q1–Q3]	19.43 [10.54–48.34]	14.31 [9.08–24.75]	69.9 [32.42–147.4]	<0.0001
**Outcomes**				
IMV, no. (%)	60 (22.47%)	15 (8.11%)	45 (54.88%)	<0.0001
Mortality, no. (%)	82 (30.71%)	-	82 (30.71%)	<0.0001
IMV + Mortality, no. (%)	45 (16.85%)	-	45 (16.85%)	<0.0001
Hospital stays, day median [Q1-Q3]	8 [6–13]	8 [6–11]	12 [6–17.75]	0.0005

AH = arterial hypertension; IHD = ischemic heart disease; AF = atrial fibrillation; CHF = chronic heart failure; MI = myocardial infarction; T2D = type 2 diabetes; COPD = chronic obstructive pulmonary disease; PAD = peripheral arterial disease; CKD = chronic kidney disease; CVA = cerebrovascular accident; PLT = total platelet count; BUN = blood urea nitrogen; MLR = monocyte to lymphocyte ratio; NLR = neutrophil to lymphocyte ratio; SII = systemic inflammatory index; SIRI = systemic inflammation response index; AISI = aggregate index of systemic inflammation; IL-6 = interleukin-6; IMV = invasive mechanic ventilation.

**Table 2:** ROC curves, optimal cut-off value, AUC, and predictive accuracy of inflammatory markers (MLR, NLR, SII, SIRI, and AISI) and TSS.

Variables	Cut-Off	AUC	Std. Error	95% CI	Sensitivity	Specificity	*p* Value
	**IMV**						
MLR NLR SII	0.54	0.783	0.033	0.717–0.848	70%	76.8%	<0.0001
	6.82	0.827	0.029	0.771–0.883	76.7%	76.3%	<0.0001
	2166.04	0.814	0.030	0.756–0.873	71.7%	79.8%	<0.0001
SIRI	3.66	0.822	0.029	0.765–0.880	90%	66.2%	<0.0001
AISI	994.76	0.813	0.030	0.754–0.871	88.3%	67.6%	<0.0001
IL-6	30.95	0.762	0.034	0.695–0.830	74.1%	75.6%	<0.0001
TSS	16.50	0.807	0.032	0.745–0.870	70%	81.2%	<0.0001
	**Mortality**						
MLR NLR SII	0.54	0.826	0.029	0.771–0.882	74.4%	81.6%	<0.0001
	6.97	0.869	0.025	0.820–0.911	80.5%	85.4%	<0.0001
	1739.36	0.845	0.026	0.794–0.896	82.9%	79.5%	<0.0001
SIRI	3.84	0.858	0.025	0.809–0.907	86.6%	73.5%	<0.0001
AISI	973.59	0.836	0.026	0.784–0.888	84.1%	71.4%	<0.0001
IL-6	28.17	0.808	0.031	0.747–0.870	77.6%	80.6%	<0.0001
TSS	15.50	0.811	0.029	0.754–0.867	73.2%	78.9%	<0.0001
	**IMV & Mortality**						
MLR NLR SII	0.55	0.842	0.032	0.780–0.905	80%	77%	<0.0001
	6.97	0.887	0.021	0.846–0.928	91.1%	76.6%	<0.0001
	2166.04	0.876	0.022	0.833–0.918	86.7%	79.3%	<0.0001
SIRI	4.70	0.892	0.020	0.852–0.931	93.3%	72.5%	<0.0001
AISI	1403.56	0.880	0.022	0.838–0.922	88.9%	75.7%	<0.0001
IL-6	30.95	0.825	0.037	0.753–0.897	89.7%	74.1%	<0.0001
TSS	16.50	0.823	0.031	0.762–0.884	73.3%	78.4%	<0.0001

AUC = area under curve; Std = standard; CI = confidence interval; MLR = monocyte to lymphocyte ratio; NLR = neutrophil to lymphocyte ratio; SII = systemic inflammatory index; SIRI = Systemic Inflammation Response Index; AISI = Aggregate Index of Systemic Inflammation; IMV = invasive mechanic ventilation; TSS = total system score; IL-6 = interleukin-6.

**Table 3:** Univariate analysis of MLR, NLR, SII, SIRI, AISI, IL-6, and TSS and all patients’ adverse event occurrences during the study period.

	IMV	Mortality	IMV & Mortality
**low-MLR vs. high-MLR**	18/170 (10.59%) vs. 42/97 (43.30%) *p* < 0.0001 OR:6.44 CI: (3.42–12.13)	21/170 (12.35%) vs. 61/97 (62.89%) *p* < 0.0001 OR:11.38 CI: (6.18–20.97)	9/170 (5.29%) vs. 36/97 (37.11%) *p* < 0.0001 OR:10.55 CI: (4.80–23.20)
**low-NLR vs. high-NLR**	14/172 (8.14%) vs. 46/95 (48.42%) *p* < 0.0001 OR:10.59 CI: (5.37–20.88)	16/174 (9.20%) vs. 66/93 (70.97%) *p* < 0.0001 OR:24.13 CI: (12.20–47.73)	4/174 (2.30%) vs. 41/93 (44.09%) *p* < 0.0001 OR:33.50 CI: (11.46–97.95)
**low-SII vs. high-SII**	17/182 (9.34%) vs. 43/85 (50.59%) *p* < 0.0001 OR:9.93 CI: (5.15–19.14)	14/161 (8.70%) vs. 68/106 (64.15%) *p* < 0.0001 OR:18.78 CI: (9.54–36.97)	6/182 (3.30%) vs. 39/85 (45.88%) *p* < 0.0001 OR:24.86 CI: (9.92–62.32)
**low-SIRI vs. high-SIRI**	6/143 (4.20%) vs. 54/124 (43.55%) *p* < 0.0001 OR:17.61 CI: (7.22–42.94)	11/147 (7.48%) vs. 71/120 (59.17%) *p* < 0.0001 OR:17.91 CI: (8.77–36.58)	3/164 (1.83%) vs. 42/103 (40.78%) *p* < 0.0001 OR:36.95 CI: (11.04–123.64)
**low-AISI vs. high-AISI**	7/147 (4.76%) vs. 53/120 (44.17%) *p* < 0.0001 OR:15.82 CI: (6.82–36.65)	13/145 (8.97%) vs. 69/122 (56.56%) *p* < 0.0001 OR:13.21 CI: (6.74–25.90)	5/173 (2.89%) vs. 40/94 (42.55%) *p* < 0.0001 OR:24.88 CI: (9.35–66.24)
**low-IL-6 vs. high-IL-6**	15/173 (8.67%) vs. 45/94 (47.87%) *p* < 0.0001 OR:9.67 CI: (4.96–18.83)	17/171 (9.94%) vs. 63/96 (65.63%) *p* < 0.0001 OR:17.29 CI: (8.98–33.27)	4/173 (2.31%) vs. 41/94 (43.62%) *p* < 0.0001 OR:32.68 CI: (11.18–95.48)
**low-TSS vs. high-TSS**	15/168 (8.93%) vs. 45/99 (45.45%) *p* < 0.0001 OR:8.50 CI: (4.38–16.47)	29/186 (15.59%) vs. 53/81 (65.43%) *p* < 0.0001 OR:10.24 CI: (5.59–18.77)	10/158 (6.33%) vs. 35/99 (35.35%) *p* < 0.0001 OR:8.09 CI: (3.77–17.33)

MLR = monocyte to lymphocyte ratio; NLR = neutrophil to lymphocyte ratio; SII = systemic inflammatory index; SIRI = Systemic Inflammation Response Index; AISI = Aggregate Index of Systemic Inflammation; IL-6 = interleukin-6; IMV = invasive mechanic ventilation; TSS = total system score.

**Table 4:** Multivariate analysis of new adverse events occurred during the entire study period.

	IMV			Mortality			IMV & Mortality		
	OR	95% CI	*p* Value	OR	95% CI	*p* Value	OR	95% CI	*p* Value
**Age > 70** **AF** **CHF**	1.97	1.07–3.63	0.02	2.49	1.43–4.35	0.001	2.87	1.38–5.96	0.005
	2.22	1.22–4.04	0.009	2.58	1.48–4.49	<0.001	2.21	1.14–4.27	0.01
	1.51	0.84–2.69	0.16	1.90	1.12–3.23	0.01	2.17	1.11–4.22	0.02
**MI**	1.35	0.73–2.48	0.33	1.55	0.89–2.71	0.11	1.36	0.69–2.67	0.37
**D** **yslipidemia**	2.13	1.15–3.96	0.01	1.93	1.12–3.32	0.01	2.16	1.08–4.35	0.02
**CKD**	1.31	0.66–2.57	0.43	2.53	1.38–4.64	0.003	2.14	1.05–4.33	0.03
**CVA**	0.89	0.46–1.70	0.72	1.74	0.99–3.05	0.052	1.32	0.66–2.62	0.42
**Unvaccinated**	1.90	1.01–3.55	0.04	3.02	1.69–5.40	<0.001	2.97	1.47–6.00	0.002
**Fully Vaccinated**	0.16	0.08–0.33	<0.001	0.46	0.27–0.79	0.006	0.23	0.11–0.51	<0.001
**high-MLR** **high-NLR** **high-SII**	6.44	3.42–12.13	<0.001	6.49	2.51–22.24	<0.001	11.85	5.37–26.14	<0.001
	10.59	5.37–20.88	<0.001	24.13	12.20–47.73	<0.001	33.51	11.46–97.95	<0.001
	9.93	5.15–19.14	<0.001	18.78	9.54–36.97	<0.001	24.87	9.92–62.32	<0.001
**high-SIRI**	17.61	7.22–42.94	<0.001	17.91	8.77–36.58	<0.001	36.95	11.04–123.64	<0.001
**high-AISI**	15.82	6.86–36.65	<0.001	13.21	6.74–25.90	<0.001	24.88	9.35–66.24	<0.001
**high-IL-6**	8.88	4.55–17.30	<0.001	14.45	7.55–27.61	<0.001	25.06	8.54–73.51	<0.001
**high-TSS**	8.50	4.38–16.47	<0.001	10.24	5.59–18.77	<0.001	8.64	4.03–18.48	<0.001

AF = atrial fibrillation; CHF = chronic heart failure; MI = myocardial infarction; CKD = chronic kidney disease; CVA = cerebrovascular accident; MLR = monocyte to lymphocyte ratio; NLR = neutrophil to lymphocyte ratio; SII = systemic inflammatory index; SIRI = Systemic Inflammation Response Index; AISI = Aggregate Index of Systemic Inflammation; IL-6 = interleukin-6; IMV = invasive mechanic ventilation; TSS = total system score.

## Data Availability

The data presented in this study are available on request from the corresponding author. The data are not publicly available due to ethical restrictions.
